# Practical consideration for successful sequential tumor biopsies in first-in-human trials

**DOI:** 10.1007/s10637-022-01236-4

**Published:** 2022-04-11

**Authors:** Takafumi Koyama, Toshio Shimizu, Jun Sato, Yuki Katsuya, Satoru Iwasa, Shunsuke Kondo, Tatsuya Yoshida, Kazuki Sudo, Makoto Nishino, Yuichi Takiguchi, Kan Yonemori, Noboru Yamamoto

**Affiliations:** 1 Department of Experimental Therapeutics, National Cancer Center Hospital, Tokyo, Japan; 2 Department of Gastrointestinal Oncology, National Cancer Center Hospital, Tokyo, Japan; 3 Department of Hepatobiliary and Pancreatic Oncology, National Cancer Center Hospital, Tokyo, Japan; 4 Department of Thoracic Oncology, National Cancer Center Hospital, Tokyo, Japan; 5 Department of Medical Oncology, National Cancer Center Hospital, Tokyo, Japan; 6 Department of Medical Oncology, Chiba University, Chiba, Japan

**Keywords:** Biopsy, Clinical Trial, Disease Progression, Diagnostic Imaging, Therapeutics/AE

## Abstract

In first-in-human (FIH) trials, sequential tumor biopsies, i.e., two consecutive tumor biopsies, the first performed at baseline (pretreatment) and the second during the early treatment period (on-treatment), provide proof of concept in investigational new drugs. We evaluated the success of sequential tumor biopsies in FIH trials, and explored approaches for improved success rates. We retrospectively reviewed the sequential tumor biopsies required in 17 of 52 FIH trials conducted from 2015 to 2020. One hundred and thirty-eight patients were identified. Success of either pretreatment or on-treatment biopsy alone, and of sequential tumor biopsies, was defined as the acquisition of viable tumor cells and as obtaining tumor cells from both biopsy specimens, respectively. The success rates of pretreatment and on-treatment biopsy were 98.6% and 94.2%, respectively, and of sequential tumor biopsies was 70.3%. Adverse events associated with the pretreatment biopsies (33.3% positive; 72.0% negative) and timing of the first imaging assessment (before on-treatment biopsy = 40.0%; after on-treatment biopsy = 82.7%) correlated with successful sequential tumor biopsies. The reasons for unsuccessful sequential tumor biopsies could be categorized into two groups: 1) patient refusal of the on-treatment biopsy (most frequently due to early disease progression); and 2) absence of tumor cells in the pretreatment or on-treatment biopsy specimen. We propose an approach to achieving greater success in sequential tumor biopsies in FIH trials; the first imaging assessment during the study should be scheduled after on-treatment biopsy. (Registration number UMIN000042487, Date of registration November 18, 2020).

## Introduction

In recent decades, sequential tumor biopsies, i.e., two consecutive tumor biopsies, the first performed at baseline and the second during the early treatment period, have often been included in phase I trials, especially first-in-human (FIH) trials, to provide proof of concept of investigational new drugs (INDs), the biologic effect of drugs on target molecules, and to assess the tumor microenvironment [1–3]. An increasing number of FIH trials have required sequential tumor biopsies in their protocols [4]. This practice has raised a number of issues, however, including safety and ethical concerns [5–7]. In the past three decades, several studies have focused on the performance of a single tumor biopsy at baseline in clinical trials and have reported a success rate (defined as an adequate number of viable tumor cells) of 70%–90% [6, 8–10]. These studies also investigated the different factors affecting successful single tumor biopsies, including biopsy site, technical procedure, size of target lesion, necrosis on imaging, and operator dependence. One study investigated the success rate of sequential tumor biopsies in clinical trials at an academic medical center [11], and reported a success rate of 41.7%. However, no study has analyzed why the success rate of sequential tumor biopsies is notably lower than that of a single tumor biopsy.

Here, we evaluated our performance in FIH trials requiring sequential tumor biopsies and analyzed the detailed reasons for unsuccessful sequential tumor biopsies. We propose an approach to achieving greater success in sequential tumor biopsies in FIH trials.

## Materials and methods

### Patient data collection

We conducted retrospective research of the 17 of 52 FIH trials that required sequential tumor biopsies at the National Cancer Center Hospital (NCCH) in Tokyo from July 2015 to December 2020. We analyzed patient characteristics, including age, gender, Eastern Cooperative Oncology Group (ECOG) performance status (PS), and cancer type. The types of IND, biopsy site, technical procedure, and biopsy outcome (tumor cell acquisition, adverse events [AEs]) were also analyzed. We also evaluated the timing of the on-treatment biopsy and the first imaging assessment during the study.

### Definition of terms

We defined the biopsy performed at baseline as the “pretreatment biopsy” and the biopsy performed during the early treatment period as the “on-treatment biopsy.” A pretreatment biopsy or on-treatment biopsy was defined as successful if the acquisition of tumor cells in the specimen was confirmed by the designated pathologist [12]. A pair of sequential tumor biopsies was defined as successful if tumor cells were obtained from both pretreatment and on-treatment biopsy specimens. This research categorized biopsy sites as follows: skin/soft tissue (skin, subcutaneous tissue, breast, and vagina), bone/internal organs (bone, pleura, peritoneum, kidney, and mediastinum), and gastrointestinal tract (stomach, esophagus, and rectum).

We calculated the success rate of sequential tumor biopsies as the number of patients in whom both the pretreatment biopsy and the on-treatment biopsy were performed successfully per the number of patients enrolled in each FIH trial. We graded biopsy-related AEs according to the NCI Common Terminology Criteria for Adverse Events version 4.0. [13].

### Statistical analysis

Univariate analysis (Fisher’s exact test) and multivariate analysis (logistic regression model) were performed to examine whether any of the following three factors affected the success of sequential tumor biopsies: ECOG PS, AEs related to pretreatment biopsies, and the first imaging assessment during the study (before vs. after on-treatment biopsy). All tests were two-sided, and a *P*-value < 0.05 was considered statistically significant. All statistical analyses were performed using commercial software (JMP version 14.3; SAS Inc., Cary, North Carolina, USA).

## Result

### Patient characteristics and biopsies

**Fig. 1:**
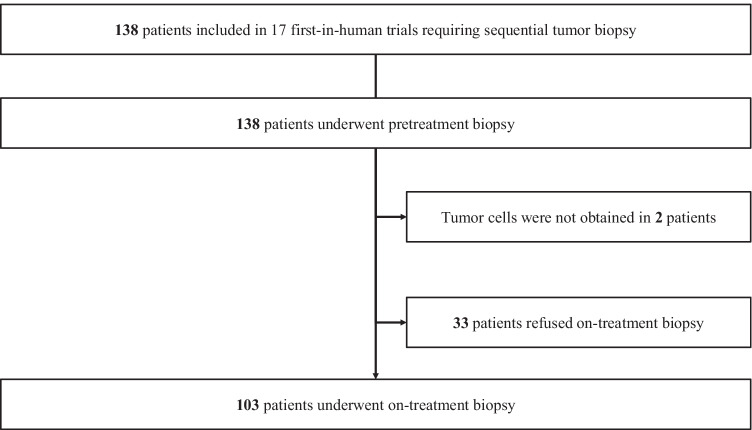
Patient flow diagram All 138 patients who were enrolled in 17 oncology first-in-human trials that required sequential biopsies underwent pretreatment biopsies. Two patients had no tumor cells in the pretreatment biopsies. Thirty-three patients refused on-treatment biopsy, with 103 patients undergoing on-treatment biopsies

**Fig. 2:**
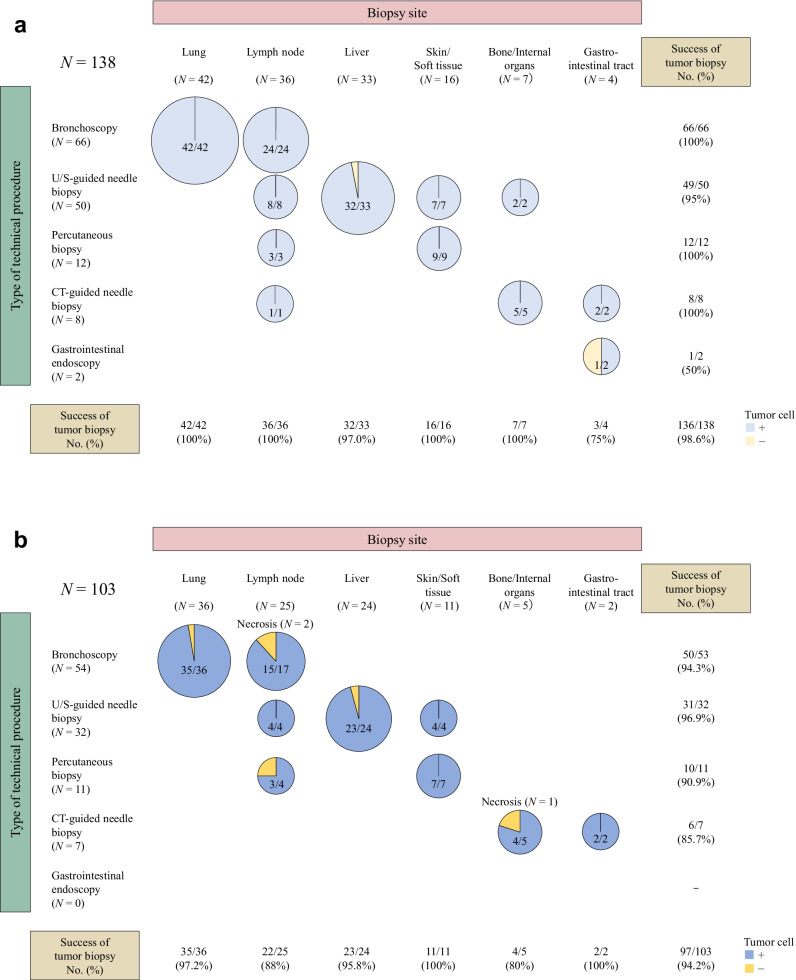
**a** Biopsy site and technical procedure in pretreatment biopsy. Each pretreatment biopsy and on-treatment biopsy were defined as successful if the acquisition of adequate tumor cells in the specimen were confirmed by the designated pathologist. This research categorized biopsy sites as follows; skin/soft tissue (skin, subcutaneous tissue, breast, and vagina), bone/internal organ (bone, pleural membrane, peritoneal membrane, kidney, and mediastinum), and gastrointestinal tract (stomach, esophagus, and rectum). The success rate of tumor biopsy sampling in the pretreatment biopsy was 98.6%. **b **Biopsy site and technical procedure in on-treatment biopsy The success rate of tumor biopsy sampling in the on-treatment biopsy was 94.2%. On-treatment biopsy specimens from six patients did not contain tumor cells, and specimens from three of these patients contained only necrotic cells, most likely due to the efficacy of the INDs revealed necrosis based on evaluation of H&E-stained slides by pathological diagnosis

A total of 138 patients were identified in the 17 FIH trials (Fig. 1). The numbers of patients who underwent pretreatment and on-treatment biopsy were 138 and 103, respectively. Patient characteristics and type of IND are shown in Table 1. In terms of cancer type, lung cancer was the most common with 70 patients, followed by colorectal cancer with 13 patients. One hundred and ten patients were enrolled in trials of immuno-oncology drugs or antibody–drug conjugates. The most common biopsy sites were lung and lymph nodes (cervical, supraclavicular, mediastinal, and hilar) in both pretreatment and on-treatment biopsies (Fig. 2a and b). The most common technical procedures were bronchoscopy and ultrasound (U/S)-guided needle biopsy in both pretreatment and on-treatment biopsies. The median size of biopsy site at baseline was 36.5 mm (25th–75th percentile: 26.7–51.2).

**Table 1:** Patient characteristics

**Characteristic**	***N*** **(%)**
**Total**	138
**Gender**	
Male/Female	77 (55.8)/61 (44.2)
**ECOG**^**a**^** Performance Status**	
0/1	81 (58.7)/57 (41.3)
**Age, years**	
> 65/ ≤ 65	35 (25.4)/103 (74.6)
**Cancer type**	
Lung cancer	60 (43.5)
Colorectal cancer	13 (9.4)
Pancreatic cancer	9 (6.5)
Ovarian cancer	7 (5.1)
Breast cancer	6 (4.3)
Melanoma	6 (4.3)
Sarcoma	6 (4.3)
Head and neck cancer	5 (3.6)
Thymic cancer	5 (3.6)
Esophageal cancer	4 (2.9)
Uterine cancer	3 (2.2)
Bile duct cancer	2 (1.5)
Kidney cancer	2 (1.5)
Neuroendocrine tumor	2 (1.5)
Stomach cancer	2 (1.5)
Other	6 (4.3)
**Type of investigational new drug**	
Immuno-oncology drug	56 (40.6)
Antibody–drug conjugate	54 (39.1)
Immuno-oncology drug and cytotoxic drug	20 (14.5)
Molecularly targeted drug	8 (5.8)

^a^*ECOG* Eastern Cooperative Oncology Group

### Performance of tumor biopsy sampling

Pretreatment biopsy specimens from two patients did not contain tumor cells (Fig. 2a). The pretreatment biopsy in one patient was performed in the liver, while the on-treatment biopsy was performed in the lung because the pretreatment biopsy specimen did not contain tumor cells. The technical procedures used in on-treatment biopsy of three patients were changed from those used in pretreatment biopsies because of AEs related to the pretreatment biopsies: from U/S-guided needle biopsy to bronchoscopy (*N* = 1), from U/S-guided needle biopsy to percutaneous biopsy (*N* = 1), and from computed tomography (CT)-guided needle biopsy to percutaneous biopsy (*N* = 1). Thirty-three patients did not undergo on-treatment biopsies. On-treatment biopsy specimens from six patients did not contain tumor cells, and specimens from three of these patients contained only necrotic cells, most likely due to the efficacy of the INDs (Fig. 2b).

Table 2 shows the details and AE rates related to pretreatment and on-treatment biopsies. The AE rates for pretreatment biopsy and on-treatment biopsy were 4.3% and 3.9%, respectively. Lung was the biopsy site with the highest frequency of AEs for both pretreatment (7.1%) and on-treatment biopsy (8.3%). Bronchoscopy was the technical procedure with the highest frequency of AEs for both pretreatment (7.6%) and on-treatment biopsies (7.5%).

**Table 2:** Adverse events related to pretreatment biopsies and on-treatment biopsies

	***N,* (%)**
**Pretreatment biopsies**	138
Pneumonia (Grade^a^ 3)	1 (0.7)
Mediastinal hemorrhage (Grade 2)	1 (0.7)
Biliary fistula (Grade 2)	1 (0.7)
Biopsy-related pain (Grade 2)	1 (0.7)
Hemoptysis (Grade 1)	2 (1.5)
None	132 (95.7)
**On-treatment biopsies**	104
Mediastinal hemorrhage (Grade 2)	1 (0.9)
Fever (Grade 1)	1 (0.9)
Hemoptysis (Grade 1)	2 (1.9)
None	100 (96.3)

^a^*Grade* Common Terminology Criteria for Adverse Events version 4.0 Grade

### Performance of sequential tumor biopsies

**Fig. 3:**
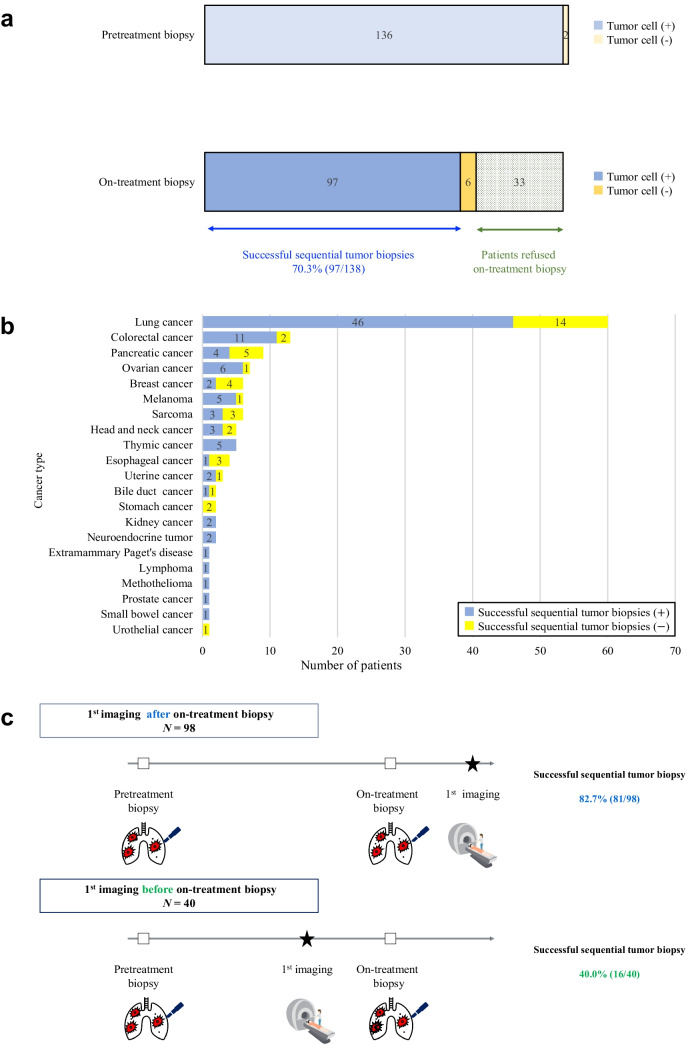
**a** Success of sequential tumor biopsy The sequential tumor biopsies were defined as successful if tumor cells were obtained from both pretreatment and on-treatment biopsy specimens. The success rate of sequential tumor biopsies was 70.3%. All 138 patients who were enrolled in 17 first-in-human trials underwent pretreatment biopsy (*N* = 138). An on-treatment biopsy was not carried out in 33 patients because of the patients’ refusal. A total of 103 underwent on-treatment biopsy, with 97 biopsies considered successful. **b** Successful sequential tumor biopsy by cancer type The number of patients who had successful sequential tumor biopsies and the number of patients who did not have successful sequential tumor biopsies by cancer type are shown. **c** Impact of 1^st^ tumor imaging timing on successful sequential tumor biopsy Of the forty-three patients undergoing the first imaging assessment before on-treatment biopsy, 16 patients subsequently underwent on-treatment biopsy (41.0%). The first imaging assessments were not performed between pretreatment biopsy and on-treatment biopsy in 95 patients. Of these 99 patients, successful sequential tumor biopsies were performed in 81 patients (81.8%)

The success rate of sequential tumor biopsies was 70.3% (95% CI = 62.2–77.3) (Fig. 3a). The number of patients who had successful and unsuccessful sequential tumor biopsies is shown according to each cancer type in Fig. 3b. Pretreatment biopsies were performed in 138 patients, and the biopsy specimens from 136 patients contained tumor cells. Thirty-three patients refused on-treatment biopsy for the following reasons: early disease progression (*N* = 21), *deteriorated* physical condition due to AEs with INDs (*N* = 8), AEs related to the pretreatment biopsies (*N* = 3) and absence of a biopsiable lesion due to tumor shrinkage (*N* = 1). On-treatment biopsies were performed in 103 patients, and the biopsy specimens from 97 patients contained tumor cells.

### Timing of the first imaging assessment on successful sequential tumor biopsies

We examined whether the timing of the first imaging assessment during the study affected successful sequential tumor biopsy in Fig. 3c. The first imaging assessments were performed before on-treatment biopsies in 40 patients (6 patients according to the protocol schedule and 34 patients for the purpose of evaluating AEs). Of these 40 patients, successful sequential tumor biopsy was performed in 16 patients (40.0% [95% CI = 26.3–55.4]). Nineteen patients refused on-treatment biopsy due to early disease progression confirmed on imaging (*N* = 17) or clinical symptoms (*N* = 2). Four patients refused on-treatment biopsy for the following reasons: *deteriorated* conditions due to AEs with INDs (*N* = 3) and mediastinal hemorrhage (Grade 2) related to the pretreatment biopsy (*N* = 1). The first imaging assessments were not performed between pretreatment biopsy and on-treatment biopsy in 98 patients. Of these 98 patients, successful sequential tumor biopsy was performed in 81 patients [82.7% (95% CI = 74.0 to 88.9)]. Ten patients refused on-treatment biopsy for the following reasons: *deteriorated* condition due to AEs from INDs (*N* = 5), early disease progression confirmed on clinical symptoms (*N* = 2), the AE of biopsy-related pain (Grade 2) and pneumonia (Grade 3) related to the pretreatment biopsy (*N* = 2), and absence of a biopsiable lesion due to tumor shrinkage (*N* = 1).

### Factors correlating with success of sequential tumor biopsies and tumor biopsy sampling

Patient characteristics, including ECOG PS, did not affect successful sequential tumor biopsy (Table 3). AEs related to pretreatment biopsy (33.3% positive; 72.0% negative) did not affect successful sequential tumor biopsy in univariate analysis (*P* = 0.0639), but did in multivariate analysis (*P* = 0.0057). The timing of the first imaging assessment during the study (before on-treatment biopsy = 40.0%; after on-treatment biopsy = 82.7%) had a major impact on successful sequential tumor biopsies in both univariate and multivariate analysis, with *P* < 0.0001 in both.

**Table 3:** Univariate and multivariate analysis of successful sequential tumor biopsy by ECOG Performance Status, adverse events related to pretreatment biopsy, and timing of the first imaging assessment

**Factor**	**Successful sequential tumor biopsy** ***N,*** **(%)**	**Univariate analysis**	**Multivariate analysis**
**ECOG**^**a**^** Performance Status**		*P* = .248	*P* = .259
0	60/81 (74.1%)		
1	37/57 (64.9%)		
**Adverse events related to pretreatment biopsy**		*P* = .0639	*P* = .0057
Yes	2/6 (33.3%)		
No	95/132 (72.0%)		
**Timing of the first imaging assessment**		*P* < .0001	*P* < .0001
Before on-treatment biopsy	16/40 (40.0%)		
After on-treatment biopsy	81/98 (82.7%)		

^a^*ECOG* Eastern Cooperative Oncology Group

## Discussion

Sequential tumor biopsies in FIH trials play a key role in providing proof of concept of the mechanism of action of INDs. The difficulty of achieving success with sequential tumor biopsies has been reported [11, 14]. However, no report has suggested approaches to overcome this difficulty. We evaluated our performance of sequential tumor biopsies in the FIH trials and explored approaches for a higher success rate.

Our research shows that the success rate of sequential tumor biopsies in FIH trials was as high as 70.3%. The fact that all patients underwent pretreatment biopsy after enrollment in the FIH trials suggests that these patients were prepared to undergo tumor biopsy sampling as required in the protocols, and that none revoked an earlier agreement to participate on learning of the protocol necessity for biopsy, as has been reported elsewhere [6, 10, 15]. In both pretreatment and on-treatment biopsies, the success rate of tumor biopsy sampling was considered to be higher than that generally reported, and the rate of biopsy-related AEs was considered lower [9, 11, 14]. AEs related to the pretreatment biopsies were negatively associated with the completion of successful sequential tumor biopsies (Table 3). The size of biopsy site at baseline was not associated to the pretreatment biopsy success (*P* = 0.1294). In our research, the minimal required size for successful sequential tumor biopsy could not be determined possibly due to the selection bias in enrolling FIH trials. In selecting biopsy site and type of technical procedure, it is important to maximize the possibility of tumor cells being obtained in the biopsy specimen, but also to minimize the possibility of biopsy-related AEs [16, 17]. The success rate of on-treatment biopsy (94.2%) was slightly lower than that of pretreatment biopsy (98.6%) (*P* = 0.0763). This was partly related to necrosis in the on-treatment biopsy specimens due to the efficacy of the IND in three patients.

There was a discordance between our success rate in sequential tumor biopsies (70.3%) and the success rate of pretreatment (98.6%) and on-treatment biopsies (94.2%), respectively. The major reason for this discordance was the patients’ refusal of on-treatment biopsy (*N* = 33), and 21 out of 33 refusal (63.6%) were related to early disease progression. On-treatment biopsies could not be performed when the patients felt distressed due to early disease progression, even though the biopsy target lesions were larger and easier to biopsy [8, 9]. The American Society of Clinical Oncology has published clear guidelines on tumor biopsy sampling in early-phase clinical trials [18], with the core ethical principles of minimizing risk for participants.

Liquid biopsy is less invasive to the patient than tissue biopsy [19, 20]. Patients would most likely be willing to consent to a blood draw, instead of a painful biopsy procedure, even in the presence of early disease progression. As the number of phase I trials in which patients can participate based on the results of liquid biopsy is increasing, liquid biopsy may be able to replace invasive tumor biopsies [21]. However, liquid biopsy faces barriers in completely replacing tissue biopsy as a means of elucidating the proof of concept of an IND, since it cannot be used to evaluate the microenvironment around the tumor [22].

The success rate of sequential tumor biopsies was significantly lower when the first imaging assessment was performed before the on-treatment biopsy (40.0%) compared to when it was performed after the on-treatment biopsy (82.7%). Patients inevitably refused on-treatment biopsies once the imaging assessments reveal early disease progression. In our research, the first imaging assessments were performed in 40 patients before on-treatment biopsies, and revealed early disease progression confirmed on imaging in 17 patients. All 17 of these patients refused on-treatment biopsies. The first imaging assessment during the study should be scheduled after on-treatment biopsy in the protocol. The first imaging assessments were actually performed before on-treatment biopsy in 6 patients according to the protocol schedule in our research. If imaging assessment was scheduled after on-treatment biopsy in the protocol, the success rate of sequential tumor biopsies could be estimated to improve from 70.3% to 74.6%. Selecting patients on similar IND protocols but on different imaging schedules would have been a technique to eliminate imaging schedule as a variable.

In conclusion, our research showed that the success rate of sequential tumor biopsy in FIH trial was as high as 70.3%. We analyzed the detailed reasons for unsuccessful sequential tumor biopsy. We propose a reasonable approach to achieve greater success in sequential tumor biopsy in FIH trials; the first imaging assessment during the study could be recommended to be scheduled after on-treatment biopsy.

## Data Availability

The datasets supporting the conclusion of this article are included within the article.
